# Autosomal recessive progeroid syndrome due to homozygosity for a *TOMM7* variant

**DOI:** 10.1172/JCI156864

**Published:** 2022-12-01

**Authors:** Abhimanyu Garg, Wee-Teik Keng, Zhenkang Chen, Adwait Amod Sathe, Chao Xing, Pavithira Devi Kailasam, Yanqiu Shao, Nicholas P. Lesner, Claire B. Llamas, Anil K. Agarwal, Prashant Mishra

**Affiliations:** 1 Division of Nutrition and Metabolic Diseases, Department of Internal Medicine, Center for Human Nutrition, UT Southwestern Medical Center, Dallas, Texas, USA.; 2 Medical Genetics Department, Kuala Lumpur Hospital, Kuala Lumpur, Malaysia.; 3 Children’s Medical Center Research Institute,; 4 McDermott Center for Human Growth and Development, and; 5 Department of Bioinformatics, UT Southwestern Medical Center, Dallas, Texas, USA.; 6 Department of Pediatrics, Hospital Pakar Sultanah Fatimah, Johor, Malaysia.; 7 Department of Pediatrics and; 8 Harold C. Simmons Comprehensive Cancer Center, UT Southwestern Medical Center, Dallas, Texas, USA.

**Keywords:** Endocrinology, Genetics, Genetic diseases, Mitochondria

## Abstract

Multiple genetic loci have been reported for progeroid syndromes. However, the molecular defects in some extremely rare forms of progeria have yet to be elucidated. Here, we report a 21-year-old man of Chinese ancestry who has an autosomal recessive form of progeria, characterized by severe dwarfism, mandibular hypoplasia, hyperopia, and partial lipodystrophy. Analyses of exome sequencing data from the entire family revealed only 1 rare homozygous missense variant (c.86C>T; p.Pro29Leu) in *TOMM7* in the proband, while the parents and 2 unaffected siblings were heterozygous for the variant. *TOMM7*, a nuclear gene, encodes a translocase in the outer mitochondrial membrane. The TOMM complex makes up the outer membrane pore, which is responsible for importing many preproteins into the mitochondria. A proteomic comparison of mitochondria from control and proband-derived cultured fibroblasts revealed an increase in abundance of several proteins involved in oxidative phosphorylation, as well as a reduction in abundance of proteins involved in phospholipid metabolism. We also observed elevated basal and maximal oxygen consumption rates in the fibroblasts from the proband as compared with control fibroblasts. We concluded that altered mitochondrial protein import due to biallelic loss-of-function *TOMM7* can cause severe growth retardation and progeroid features.

## Graphical abstract

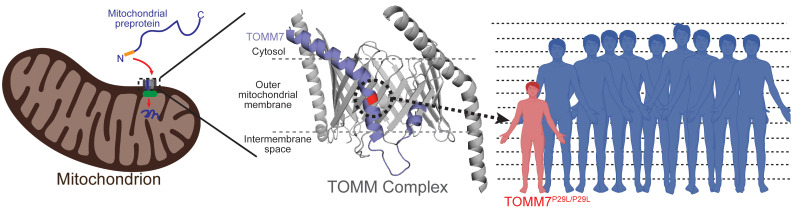

## Introduction

In the last 2 decades, considerable progress has been made in identifying the molecular genetic basis of several progeroid syndromes, including mandibuloacral dysplasia (MAD), mandibular hypoplasia, deafness and progeroid syndrome (MDPS), Hutchinson-Gilford progeria syndrome (HGPS) and atypical progeroid syndrome (APS). Disease-causing variants in *LMNA*, *ZMPSTE24*, and *MTX2* have been linked to MAD, HGPS, and APS, and variants in *POLD1* have been linked to MDPS (1). However, the molecular defects in some other extremely rare forms of progeria have yet to be identified. Here, we elucidated the genetic basis of a rare, autosomal recessive form of progeroid syndrome that presented with severe dwarfism, mandibular hypoplasia, hyperopia, micro-ophthalmia, and partial lipodystrophy. We identified the causative mutation of this disorder, a homozygous, rare missense variant in *TOMM7*, a gene that encodes a translocase of the outer mitochondrial membrane.

## Results and Discussion

A 21-year-old man of Chinese ancestry living in Malaysia was referred to UT Southwestern with a presumptive diagnosis of MAD. He was born full term and weighed 2.8 kg at birth. He had severe postnatal growth retardation and presented at age 6 with short stature and dysmorphism. His hearing was normal but his vision was poor. Although he communicated well verbally, he had significant learning disabilities.

**Figure 1:**
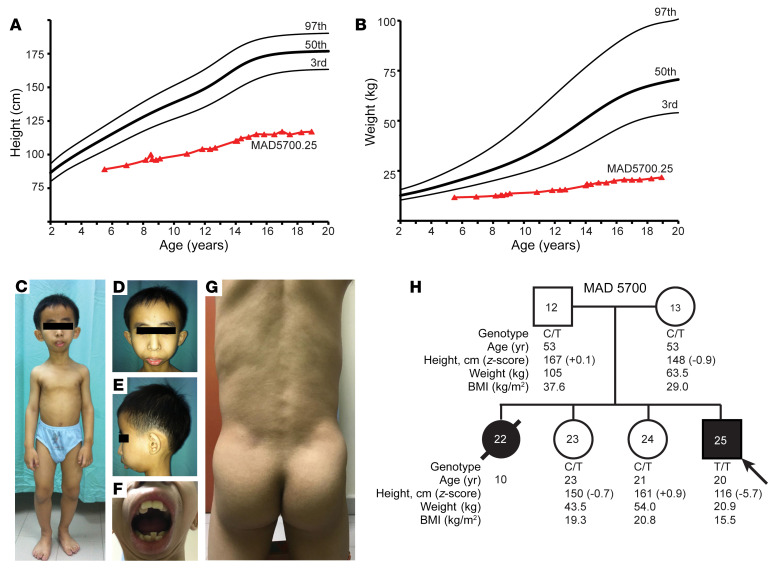
Growth charts, clinical pictures and pedigree of the patient. (**A**) Height of proband from age 5–19 years, shown as triangles, compared to normal values shown as the third, fiftieth, and ninety-seventh percentiles. (**B**) Body weight of proband from age 5–19 years, shown as triangles, compared to normal values from the Centers for Disease Control shown as the third, fiftieth, and ninety-seventh percentiles. (**C**) Anterior view of the proband at 12 years of age, showing proportionate short stature, small mandible, and muscular extremities. (**D**) Anterior view of the proband’s face, showing recession of scalp hair from the frontal region and small mandible with protruding maxillary central incisors. (**E**) Lateral view of the proband’s face, showing marked recession of the chin indicative of mandibular hypoplasia. (**F**) Anterior view of the proband’s mouth, showing crowding of both the maxillary and mandibular teeth. (**G**) Posterior view of the trunk and gluteal region of the proband showing marked muscularity and loss of subcutaneous fat indicative of lipodystrophy. (**H**) MAD 5700 pedigree with genotype and phenotype data. Circles denote females, and squares denote males. Symbols filled with black represent affected individuals with the progeroid syndrome, whereas white symbols indicate unaffected individuals. The number in the center of the symbol indicates pedigree number. Slanted arrow indicates the proband. Diagonal line across the symbol indicates deceased individual. The genotypes, age, height, weight, and body mass index (BMI) of the individuals are given under the symbols. Individuals with heterozygous c.86C>T *TOMM7* variant are indicated with “C/T,” and the proband with the homozygous variant is indicated with “T/T.”

On physical examination, his height was 116 cm, with a weight of 20.9 kg (Figure 1, A and B), and a head circumference of 53.5 cm, which were all below the third percentile when compared with age- and sex-matched controls. He had proportionate short stature with relative macrocephaly (Figure 1, C–G). He had triangular facies with broad forehead, prominent nasal bridge, bulbous nose, severe mandibular hypoplasia, and dental crowding (Figure 1, C–G). He had pendular nystagmus and high hyperopia with visual acuity of 3/60 in both eyes (right eye: +10.75 Diopter Sphere and left eye: +6.00 Diopter Sphere). He also had bilateral micro-ophthalmia with axial lengths of only 16.8 mm (Supplemental Figure 1; supplemental material available online with this article; https://doi.org/10.1172/JCI156864DS1). He had some café au lait spots on his limbs, coarse eyebrows, and sparse hair. His muscles were well defined, especially on the hips and lower extremities (Figure 1G). Most of the skinfold thickness measurements over the trunk and thighs were below the tenth percentile for normal males (Supplemental Figure 2) (2). At the age of 17 years, he reached Tanner pubertal stage V, but had sparse moustache and axillary hair.

His complete blood counts, serum sodium, potassium, chloride, calcium, phosphorus, magnesium, urea, creatinine, total protein, albumin, globulin, bilirubin, creatine kinase, and lactate dehydrogenase were within normal range. His serum alkaline phosphatase concentrations were slightly high, but serum fasting glucose, total cholesterol, triglycerides, high-density lipoprotein cholesterol, alanine aminotransferase, aspartate aminotransferase, TSH, total thyroxine, insulin-like growth factor 1 (IGF-1), LH, FSH, and testosterone concentrations were normal (Table 1).

At age 11, echocardiography showed mild mitral regurgitation with normal chamber sizes and a left ventricular ejection fraction of 73%. At age 17, echocardiography revealed mild dilatation of the left ventricle. No ventricular or atrial septal defects or patent ductus arteriosus was observed. Ultrasound of the abdomen and magnetic resonance imaging of the brain revealed no abnormalities. Skeletal surveys conducted at 11 and 17 years of age revealed no significant delay in his bone age (Supplemental Figure 3).

**Figure 2:**
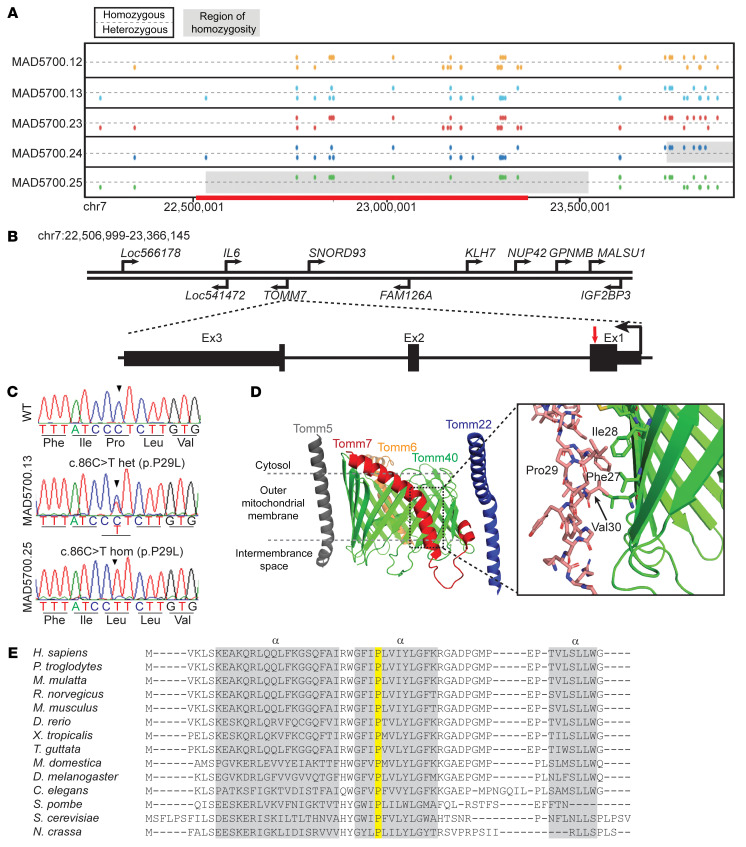
Identification of a Pro29Leu variant in the TOMM7 gene. (**A**) Schematic of segments on chromosome 7 of the proband (MAD5700.25) and his parents (MAD5700.12, 13) and sisters (MAD5700.23, 24), based on GRCh37/hg19 coordinates. For each individual, the top line displays markers with homozygous genotypes and the bottom line displays markers with heterozygous genotypes. The homozygous regions inferred from WES data are shown in gray (~1 Mb) and those derived from WGS (~0.8 Mb, chr7:22,506,999-23,366,145) shown with red. The two siblings of the proband and the parents did not share the homozygous region. (**B**) The location of various genes in the homozygous region on chromosome 7, the gene structure of *TOMM7*, and the location of the mutation in the proband. Human *TOMM7* contains three exons (shown in black rectangles) and two introns (shown as a line). The pathogenic variant c.86C>T in *TOMM7* is located in exon 1 (red arrow). (**C**) Sequence electropherogram for WT (top) sequence in exon 1 of *TOMM7*, MAD5700.12 (middle) showing heterozygous (het) c.86C>T variant, and MAD5700.25 (bottom) showing the homozygous (hom) c.86C>T variant. (**D**) An overview of the cryo-electron microscope structure for human TOM complex (PDB ID 7CK6). TOMM7 is shown in red, TOMM5 in gray, TOMM6 in orange, TOMM22 in blue, and TOMM40 in green. The expanded view shows the region surrounding residue proline (Pro) 29 in TOMM7, with nearby amino acids phenylalanine (Phe) 27, isoleucine (Ile) 28, and valine (Val) 31 forming interactions with TOMM40 (green). (**E**) Protein alignment of TOMM7 from the indicated organisms. Note that Proline 29 (highlighted in yellow) is conserved amongst all the phyla aligned. The three α-helices are indicated by gray bars.

His parents denied any consanguinity. His eldest sister also had dwarfism, sparse hair, high hyperopia and died at age 10 during a febrile illness associated with cough and shortness of breath. He has 2 older sisters who are healthy (Figure 1H). We performed whole exome sequencing (WES) on the proband, unaffected sisters, and their parents (Figure 2A). We hypothesized an autosomal-recessive inheritance and filtered for rare missense, nonsense, splicing, or frame shift variants — either homozygous or compound heterozygous — in the proband, but not in the 2 unaffected siblings or their parents. We used a liberal cutoff for variants using the minor allele frequency (MAF) < 0.01 in the genome aggregation database (gnomAD; http://gnomad.broadinstitute.org/), even though this is a rare syndrome. Other criteria included a Genomic Evolutionary Rate Profiling ++ (GERP++) score (3) greater than 2.0, and a Combined Annotation Dependent Depletion (CADD) score (4) greater than 15. There was only 1 variant that passed the filtering strategy. This was a homozygous variant, (rs778567973) in *TOMM7* (NC_000007.13:g.22862313G>A; NM_019059.4:c.86C>T; NP_061932.1:p.Pro29Leu) in the proband, and both the parents and the 2 unaffected siblings were heterozygous for the variant (Figure 2, B and C; see Supplemental methods). Among the world-wide population, heterozygotes of this variant have been seen only in Koreans, Japanese, and other East Asians with a MAF of 0.000048 (gnomAD, v2.1.1). The variant had a GERP++ score of 6.07 and CADD score of 34.

Kinship analysis using WES data confirmed that the parents were more distantly related than third-degree relatives. An approximately 1.0 MB region of homozygosity that included *TOMM7* on chromosome 7 was present in the proband, but not in any other family member (Figure 2, A and B). Sanger sequencing (Supplemental Methods) confirmed that the p.Pro29Leu variant in *TOMM7* cosegregated with the phenotype in the family (Figure 1H). We further performed whole genome sequencing (WGS) of the proband’s DNA (Supplemental Methods), which did not identify any copy number variants or rare intronic variants in the regions of homozygosity across the proband’s genome. No pathogenic variants were found in the proband in progeroid or MAD syndrome genes, including *LMNA*, *ZMPSTE24*, *BANF1*, *RECQL2*, *RECQL4*, *BLM*, *POLD1*, *POLR3A*, *WRN*, *ERCC3*, *ERCC4*, *ERCC5*, *ERCC6*, *ERCC8*, *TERT*, *TERC*, *DKC1*, *AKT1*, *SPRTN*, *XPA*, *XPC*, or *MTX2* (5, 6). None of these genes lies in a region that is homozygous in the proband but heterozygous in other family members.

TOMM7 is a protein made of 55 amino acids and is highly conserved from yeast to mammals; the proline at position 29 is completely conserved among mammals and other species (Figure 2E) (7–9). Expression databases reveal that *TOMM7* mRNA and protein are detected in many tissues (10, 11). A structure of the core human TOMM complex was recently reported (12). TOMM7 is primarily composed of a long kinked α-helix that makes multiple contacts with the TOMM40 pore protein (Figure 2D). Proline 29 breaks an α-helix in the kinked transmembrane segment of the protein (Figure 2D) and is predicted to interact with the core-channel proteins, including TOMM40 (12).

**Figure 3:**
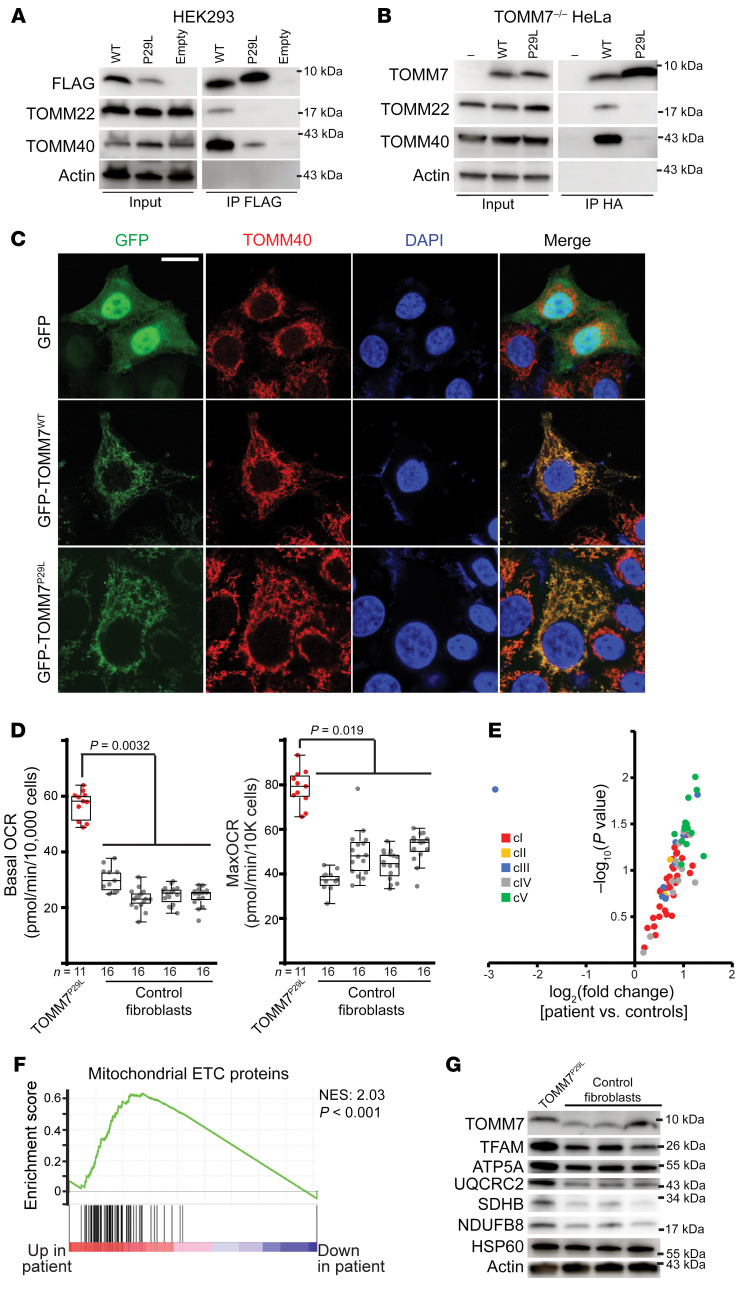
The functional impact of TOMM7 variant, P29L. (**A**) Immunoprecipitation of FLAG-TOMM7^WT^ (WT), FLAG-TOMM7^P29L^ (P29L), or empty vector transiently expressed in HEK293 cells, as assessed by Western blot. Input whole cell lysates and immunoprecipitated fractions (IP FLAG) are shown. TOMM7^P29L^ displays decreased interaction with the core TOMM complex (TOMM40 and TOMM22). Molecular weights are indicated. (**B**) Similar to **A**, but in *Tomm7^–/–^* HeLa cells transduced with TOMM7^wt^ or Tomm7^P29L^-expressing lentiviral particles. (**C**) Heterologous expression of TOMM7^WT^ (wildtype) or TOMM7^P29L^ (tagged with GFP [green]) in *Tomm7^–/–^* HeLa cells indicates both proteins are localized to mitochondria; mitochondria visualized with anti-TOMM40 (red). (**D**) Increased basal and maximal (uncoupled) oxygen consumption rates (OCR) measured in proband (*TOMM7^P29L^*) and control fibroblast cell lines. *n* = 11–16 for each cell line. A linear mixed model (GraphPad Prism) was fit to test the difference between proband and control cell lines. Box plots indicate median and interquartile values; whiskers are plotted using the Tukey method. (**E**) Volcano plot of mitochondrial ETC component protein abundance in proband versus control fibroblasts. Individual proteins are color-coded based on the mitochondrial complex with which they are associated. (**F**) GSEA of mitochondrial ETC components in proband and control cell lines. The normalized enrichment score (NES) and *P* value are indicated. (**G**) Western blot analysis of TOMM7 and candidate mitochondrial ETC proteins in proband and control cell lines. Molecular weights are indicated. Actin and HSP60 levels are shown as loading controls.

To test the interaction of the TOMM7^P29L^ variant with other core-channel proteins, we performed immunoprecipitation studies of heterologously expressed TOMM7 WT (TOMM7^wt^) or TOMM7^P29L^ in human embryonic kidney 293 (HEK293) cells. The TOMM7^P29L^ protein interacted poorly with the core TOMM complex proteins — TOMM40 and TOMM22 — in contrast to TOMM7^wt^ (Figure 3A). We replicated these results in CRISPR-Cas9 generated *TOMM7^–/–^* HeLa cells reconstituted with either HA-TOMM7^wt^ or HA-TOMM7^P29L^ (Supplemental Figure 4, A–C) and observed reduced interactions between TOMM7^P29L^ and TOMM40/TOMM22 (Figure 3B). This effect was not due to mislocalization, as GFP-tagged TOMM7^wt^ and TOMM7^P29L^ both localized to mitochondria (Figure 3C).

To assess the effects of the TOMM7^P29L^ variant, we turned our attention to dermal fibroblasts cultured from the proband and age-matched controls (see Supplemental Methods). The levels of TOMM7 protein and the morphology of the mitochondria were similar in cultured dermal fibroblasts from the proband and controls (Supplemental Figure 5A and Figure 3G). TOMM7 has been previously implicated in Parkin-induced mitophagy (13, 14). However, in the presence of the mitochondrial uncoupler carbonyl cyanide m-chlorophenylhydrazone, Parkin translocation to mitochondria and mitochondrial clearance were not impaired in the patient’s fibroblasts (Supplemental Figure 5, B and C), indicating that the P29L variant may not interfere with mitophagy.

We assessed mitochondrial respiration in the proband’s fibroblasts, making use of extracellular flux assays, which indicated elevated basal and maximal oxygen consumption rates compared with a panel of control cell lines (Figure 3D). Given that the TOMM complex facilitates the import of proteins into mitochondria, we compared the proteome of mitochondria purified from the control and patient-derived fibroblasts via label-free proteomics. Of the 587 detected mitochondrial proteins, 91 were differentially expressed (*P* < 0.05), indicating that a subset of the mitochondrial proteome is affected by the presence of the TOMM7^P29L^ variant (Supplemental Figure 5D and Supplemental Table 1). Gene set enrichment analysis (GSEA) of significantly altered proteins (*P* < 0.05) indicated a higher abundance of proteins related to ATP-production and proton transport, and reduced expression of proteins related to phospholipid metabolic pathways in the patient’s mitochondria (Supplemental Figure 5E and Supplemental Table 1). We therefore assessed the levels of mitochondrial electron transport chain (ETC) proteins in our proteomics data set. These were often upregulated in the patient’s mitochondria (Figure 3E), which we verified through GSEA (Figure 3F) and Western blot (Figure 3G), specifically TFAM, ATP5A, UQCRC2, SDHB, and NDUFB8. We corroborated these results in primary tail fibroblasts from 2-week-old genetically engineered *Tomm7^P29L/P29L^* mice (Supplemental Figure 6, A and B), which also revealed increased oxygen consumption rates (Supplemental Figure 6C). Proteomic analysis from mouse fibroblasts also revealed a tendency toward upregulation of mitochondrial ETC components in *Tomm7^P29L/P29L^* cells (Supplemental Figure 6D and Supplemental Table 1), which was verified by GSEA (Supplemental Figure 6E).

In mammals, TOMM7 is part of the TOMM complex, which contains a central pore-forming protein (TOMM40) surrounded by additional proteins (TOMM5, TOMM6, TOMM7, and TOMM22), and accessory subunits (TOMM20, TOMM70). TOMM7 facilitates transport of preproteins into mitochondria (15). Interestingly, while several genetic syndromes are caused by mutations in proteins involved in translocase of the inner mitochondrial membrane complex (16), so far only a few de novo variants in *TOMM70* have been identified to cause neurological impairment (17). This study, therefore, is the first that we know of to report of a phenotype associated with a biallelic missense variant in *TOMM7*.

Unlike what has been previously reported (18), we found that inactivation of TOMM7 was associated with changes in the concentration of selected proteins in the mitochondria of fibroblasts, including increases in several proteins involved in oxidative phosphorylation. Correspondingly, we observed elevated basal as well as maximal oxygen consumption rates in the presence of the TOMM7^P29L^ variant, suggesting that TOMM7^wt^ may constitute a negative regulator of import for a subset of the mitochondrial proteome. We also observed reduced abundance of proteins involved in phospholipid metabolism. Given that defects in enzymes involved in phospholipid biosynthetic pathways, such as AGPAT2, PIK3R1, and PCYT1A, cause human lipodystrophies (19–21), it is possible that the lipodystrophy in our patient may be related to downregulation of these pathways. Future work to assess the precise consequences of this variant on the efficiency of mitochondrial protein import may further pinpoint the specific role of TOMM7 in human physiology.

Mice with homozygous deletion of *Tomm7* also recapitulate some phenotypic features of our patient and have small body size and low body weight (22). Selective inactivation of *tomm7* in zebrafish resulted in impaired cerebrovascular network formation and cerebral hemorrhages (22), and *Tomm7* KO mice also experienced cerebrovascular abnormalities and premature death by day 21 (22). While the affected elder sister of our proband died at an early age of 10 years, the proband is doing well at 21 years of age and, based on MRI, has no structural abnormality of the brain.

TOMM7 is required for Pink1 stabilization and has been recently implicated as a positive regulator of Parkin translocation in the mitophagic response to uncoupled mitochondria (13, 14). Although the TOMM7^P29L^ variant disrupts TOMM7’s interaction with the TOMM complex, Parkin-dependent mitophagy is not significantly impaired. Consistent with this, our patient has not developed any signs of Parkinson’s disease thus far. Therefore, the role of TOMM7 in Parkin-dependent mitophagy seems to be independent of its interaction with the outer mitochondrial import pore proteins.

In summary, we report a patient who presents with severe dwarfism, mandibular hypoplasia, hyperopia and partial lipodystrophy with a biallelic inactivating missense variant in *TOMM7* that is associated with increased mitochondrial oxygen consumption.

## Methods

Details are described in the Supplemental Methods.

### Study approval.

The protocol was approved by the IRB of UT Southwestern and all participants provided written, informed consent including that for patient pictures appearing in the manuscript. Animal studies were approved by the UT Southwestern IACUC.

## Author contributions

AG, AKA, CX, and PM conceived the research, designed and supervised experiments, acquired and interpreted data and wrote the manuscript; WTK and PDK helped in phenotyping the patient; ZC, NPL, and CBL conducted experiments and acquired data; CX, AAS, and YS analyzed whole exome sequencing, whole genome sequencing, and proteomics data.

## Supplementary Material

Supplemental data

Supplemental table 1

## Figures and Tables

**Table 1:**
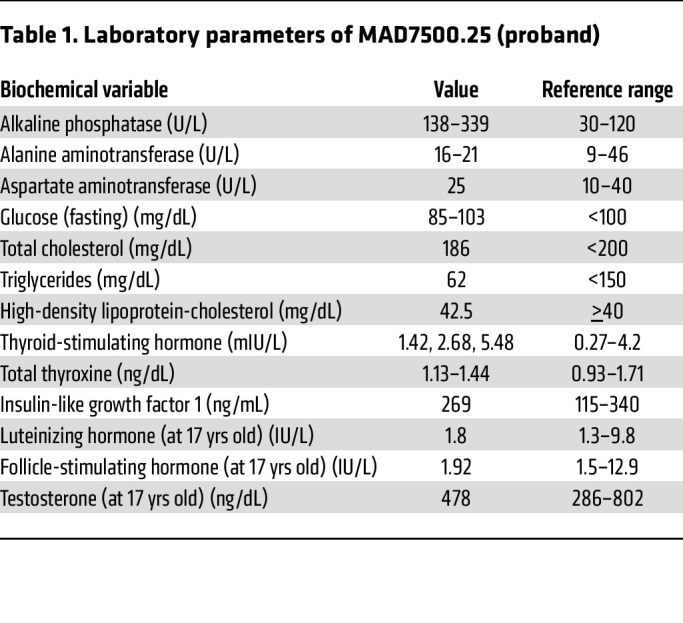
Laboratory parameters of MAD7500.25 (proband)

## Version 1 10/25/2022

In-Press Preview

## Version 2 12/01/2022

Electronic publication
